# Draft genome sequence of *Exiguobacterium* sp. strain MMG028 isolated from a salt marsh

**DOI:** 10.1128/mra.00116-23

**Published:** 2024-02-15

**Authors:** Morgan V. Farrell, Mina Y. Airkin, Tatyana N. Ali, Zainalabdin S. Altoblani, Chynna R. Bowman, Abigail Anne B. Diaz, Paul F. Faurot, Joshua E. Frausto, Sazan F. Haji, Basma A. Hamad, James B. Lively, Daniella Corene C. Luistro, Yvette Macias, Steffy Mathew, Kayla M. McKinley, Somayeh Nasirimoseloo, Bradley P. Tran, Amanda N. Trinh, Nicholas J. Shikuma

**Affiliations:** 1 Department of Biology, Viral Information Institute, San Diego State University, San Diego, California, USA; Montana State University, Bozeman, Montana, USA

**Keywords:** *Exiguobacterium*, bioremediation

## Abstract

Here, we report the draft genome sequence of *Exiguobacterium* sp. strain MMG028, isolated from Rose Creek, San Diego, CA, USA, assembled and analyzed by undergraduate students participating in a marine microbial genomics course. A genomic comparison suggests that MMG028 is a novel species, providing a resource for future microbiology and biotechnology investigations.

## ANNOUNCEMENT

To engage undergraduates in discovery-based research, novel marine bacteria were isolated and cultured. The bacterial genomes were sequenced, assembled, annotated, and analyzed by students in a marine microbial menomics (MMG) course at San Diego State University. Strain MMG028 was isolated from the water of Rose Creek Salt Marsh in San Diego, CA, USA (32.84022°N, 117.2220°W), on 30 August 2022 using a sterile cotton swab. A single colony was obtained on Marine Agar 2216 (BD Difco, Franklin Lakes, NJ, USA) and incubated at 28°C for 72 hours. Colonies were transferred to Marine Broth 2216 and incubated for 72 hours at 25°C before storage and DNA isolation.

Genomic DNA was extracted using a Quick-DNA Fungal/Bacterial Miniprep Kit (Zymo Research, Irvine, CA, USA). 16S rRNA gene amplification with primers 27F-1492R (1) and Sanger sequencing (Eton Biosciences, San Diego, CA, USA) identified the closest strain as *Exiguobacterium* sp. (94.98% ID, 0.0 E-value). DNA was submitted to the Microbial Genome Sequencing Center (Pittsburgh, PA USA) for library preparation (Illumina DNA Prep kit; San Diego, CA, USA) and whole-genome sequencing (NextSeq 550; Illumina), producing 2 × 150 bp paired-end reads (4.2M reads). Reads were trimmed using Trim Galore v.0.6.5 (2), assembled using Unicycler v0.4.8 (3), integrated into PATRIC v3.6.12 (4), and annotated using the NCBI Prokaryotic Genome Annotation Pipeline (PGAP) v5.1 (5), with default parameters. MMG028 has a 2.85 Mb genome, a total GC content of 47.75% with 16 contigs at 360.97× coverage, and an N_50_ value of 664,045 bp, with 3,015 predicted coding sequences. Default parameters were used except where otherwise noted.

**Fig 1:**
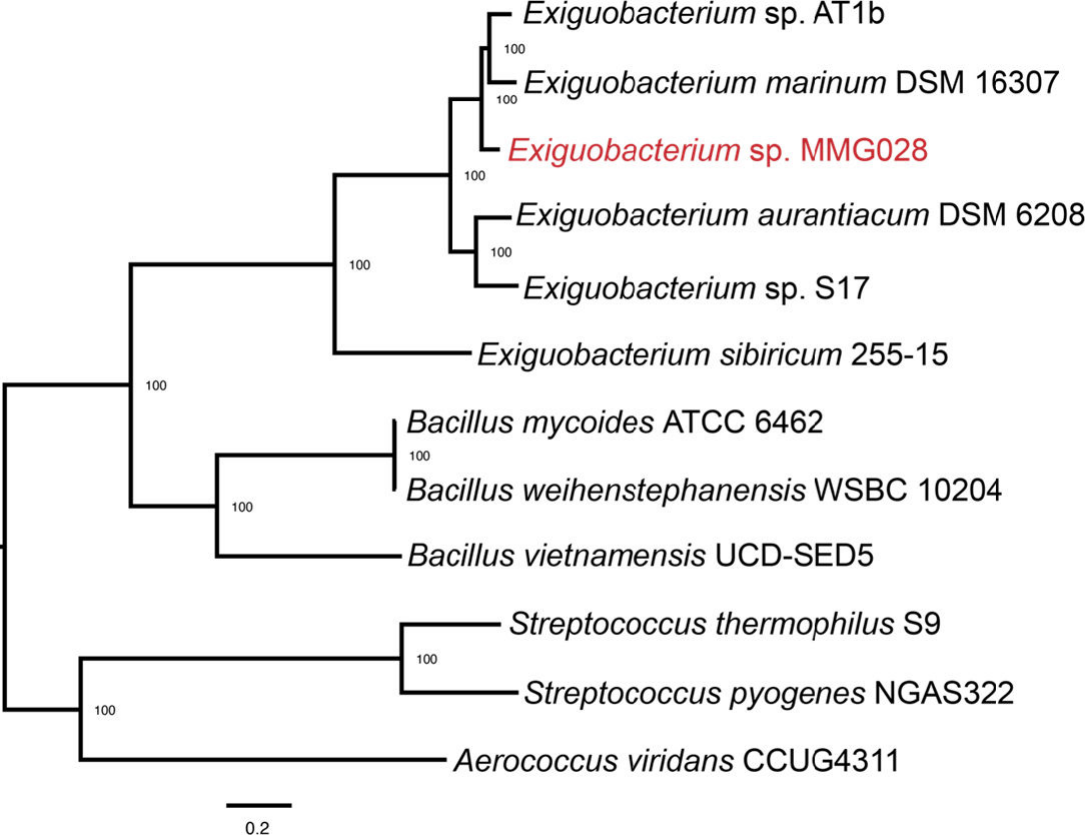
A maximum likelihood phylogeny constructed using the Codon tree method through PATRIC using 100 single-copy genes and proteins identified by PGFams (10, 12–18). The newly identified strain MMG028 is indicated in red. GenBank Accession numbers for the phylogenetic tree are as follows: *Streptococcus thermophilus* strain S9 (CP013939), *Streptococcus pyogenes* strain NGAS322 (CP010449), *Aerococcus viridans* strain CCUG4311 (CP014164), *Exiguobacterium aurantiacum* DSM 6208 (JNIQ00000000), *Exiguobacterium marinum* DSM 16307 (JHZT00000000), *Bacillus mycoides* ATCC 6462 (CP009692.1, CP009689.1, CP009691.1), *Bacillus vietnamensis* strain UCD-SED5 (LIXZ00000000), *Exiguobacterium sibiricum* 255-15 (CP001022, CP001024, CP001023), *Exiguobacterium* sp. AT1b (CP001615), *Exiguobacterium* sp. S17 (ASXD00000000), *Bacillus weihenstephanensis* strain WSBC 10204 (CP009746).

A phylogenetic analysis revealed that strain MMG028 falls into the genus *Exiguobacterium* (Fig. 1), in the family *Bacillaceae* and class *Bacilli* (6–8). Comparing strain MMG028 with *Exiguobacterium* sp. strain AT1b yields an ANI value of 88.03 (4, 9, 10), a distance that is below the 95% threshold that delineates species (11), suggesting that MMG028 is a new species. We designate the current isolate as *Exiguobacterium* sp. strain MMG028.

Species within and related to the *Exiguobacterium* genus have been found to adapt to diverse environments with the ability to withstand wide ranges of temperature, salinity, and pH (7). Features of other *Exiguobacterium* sp. strains include biosorption properties of heavy metals and other pollutants (19, 20). Strains isolated from marine environments have shown that the formation of a biofilm may be a key process for sequestering heavy metals (21). Analyses of *Exiguobacterium* sp. MMG028 with PATRIC v3.6.12 revealed several pathways involved in xenobiotic degradation including toluene, xylene, styrene, tetrachloroethene, and naphthalene (22, 23). As heavy metals and pollutants are accumulating in the environment due to human-induced industrial processes, the isolation and genome sequence of *Exiguobacterium* sp. MMG028 provides a new strain that may be a candidate for bioremediation.

## Data Availability

The genome sequencing and assembly project for strain MMG028 has been deposited in DDBJ/EMBL/GenBank under BioProject number PRJNA716944, raw sequencing SRA accession number SRX19033163, and whole-genome sequencing genome accession number JAQJIN000000000.
